# Acid sphingomyelinase modulates anxiety-like behavior likely through toll-like receptor signaling pathway

**DOI:** 10.1186/s13041-025-01178-x

**Published:** 2025-02-04

**Authors:** Huiqi Yuan, Yanan Xu, Hailun Jiang, Meizhu Jiang, Luofei Zhang, Shifeng Wei, Cao Li, Zhigang Zhao

**Affiliations:** 1 Department of Pharmacy, Beijing Tiantan Hospital, Capital Medical University, Beijing, China; 2 Department of Medicinal Chemistry, College of Pharmaceutical Sciences, Capital Medical University, Beijing, China

**Keywords:** Acid sphingomyelinase, Anxiety, Neuroinflammation, Toll-like receptor signaling pathway

## Abstract

Recent studies have shown that abnormal activity of acid sphingomyelinase (Asm) has been associated with a range of psychiatric disorders including schizophrenia and depression. However, the role of Asm in the regulation of anxiety remains unclear. In the present study, we employed Asm-knockout (Asm KO) mice to investigate the association between Asm and anxiety using behavioral tests, RNA sequencing, q-PCR, immunohistochemical staining, and other methods. The behavioral results showed that Asm KO mice exhibit enhanced anxiety-like behaviors, such as restricted activity, reduced cumulative times in the central area, diminished exploratory interest, delayed latency to feed, through behavioral tests including open field, novelty-suppressed feeding test, elevated plus maze test, ect. Transcriptional profiling combined with bioinformatics analysis revealed the upregulation of Toll-like receptor signaling pathway related gene including Tlr1/2, Ccl3, Ccl4, Ccl5 and Cd86 in Asm KO mice, which was further confirmed by the detection of activated microglia and astrocytes through iba-1 and GFAP immunohistochemical staining. Collectively, our findings uncover a role for Asm in regulating anxiety-like behavior and suggest that it may be essential for the maintenance of emotional stability, indicating its potential as a promising target for treating anxiety disorders.

**Supplementary Information:**

The online version contains supplementary material available at 10.1186/s13041-025-01178-x.

## Introduction

Psychiatric disorders represent a significant portion of the global medical, societal and economic burden, with an estimated 970 million cases in 2019 [1]. Psychiatric disorders cover a broad spectrum of classifications including depression, anxiety disorders, bipolar disorder, and schizophrenia, etc. Anxiety disorder is one of the most common mental disorders and characterized by excessive fear and anxiety, or avoidance of perceived threats, which are persistent and can impair a person’s normal life [2], and they frequently exist alongside a range of psychiatric comorbidities which further complicates their treatment and management [3]. In recent years, there has been a significant surge in the prevalence of anxiety disorders, affecting over 2.6 billion individuals worldwide [4]. In addition to its detrimental effects on mental health, anxiety also elevates the risk of various physical illnesses including stroke, diabetes, arthritis, and lung disease [5]. The widespread prevalence and complexity of anxiety disorders emphasizes their significance as a crucial public health concern, requiring continuous research to gain a deeper understanding of their intricate pathogenesis and to develop more impactful interventions.

Current research on the pathogenesis of anxiety disorders remains inconclusive and can be interpreted through various theoretical frameworks like neurotransmitter dysfunction, neuroendocrine dysfunction, immune dysfunction and neurotrophic factor dysfunction [6]. Furthermore, in recent years, neurolipids emerged as a new target for understanding the pathogenesis of anxiety. Sphingomyelin (SM) is one of the major sphingolipids, accounting for 2 to 15% of total phospholipids in mammalian cells [7]. SM is a predominant constituent of neuronal membranes and myelin, playing a crucial role in the initial development of neurons and serving as a significant regulator of synaptogenesis. Moreover, SM collaborates with cholesterol to form specific lipid raft domains within the plasma membrane, that serve as platforms for various signal transduction cascades [8]. Consequently, alterations in sphingomyelin metabolism are intricately linked to the onset and progression of neurological disorders. Currently, numerous studies have been conducted on the correlation between sphingomyelin metabolism and neurological disorders such as depression and schizophrenia [9]. However, there has been relatively limited focus on anxiety disorders. An animal study found that SM 36:1 was found to be upregulated in the dorsal hippocampus of mice exhibiting high anxiety-related behavior compared to those with low anxiety-related behavior [10]. While a study involving human participants found an inverse correlation between anxiety symptoms and serum concentration of SM 23:1 [11]. Hence, additional research is warranted to further investigate the involvement of sphingomyelin metabolism in anxiety disorders.

Numerous enzymes engage in the intricate metabolism of sphingomyelin. Among them, acid sphingomyelinase (here referred to as ASM for the human enzyme and Asm for the rodent isoform) is a significant sphingolipid-metabolizing enzyme, which hydrolyzing sphingomyelin to ceramide. In addition to its role in regulating sphingomyelin metabolism, acid sphingomyelinase is intricately linked to numerous pathological processes, including inflammation. ASM plays a pivotal role in diverse cellular contexts, releasing pro-inflammatory factors and contributing to the inflammatory cascade. Indeed, there is already some evidence for ASM in relation to other psychiatric disorders, such as depression [12, 13] and schizophrenia [14], but its role in anxiety remains unclear.

Given its role in regulating sphingomyelin metabolism and inflammation, both of which are implicated in the pathogenesis of anxiety, further investigation into the role of acid sphingomyelinase in anxiety disorders is warranted. Therefore, the present study aimed to investigate the impact of Asm on anxiety using Asm-deficient mice. Furthermore, through RNA sequencing and subsequent bioinformatics analysis, we sought to identify key genes underlying Asm related anxiolytic behaviors, which may provide a treatment strategy and therapeutic target to regulate anxiety symptoms.

## Materials and methods

### Mice

Asm-knockout (Asm KO) mice were acquired from breeding pairs of ASM heterozygous (+/-) C57BL/6J mice at the age of 8–12 weeks. Genotyping was verified by PCR analysis before the initiation of experiments. All mice were maintained in a pathogen-free environment with room temperature (22 ± 2℃) under an alternating 12:12 h light: dark cycle; food and water were available ad libitum. Male and female Asm KO mice, along with their corresponding wild-type (WT) littermates, were studied in gender-balanced designs at the age of 2 months (2 m), 3 months (3 m) and 4 months (4 m). Ethical approval for all animal procedures was obtained from the Beijing Neurosurgical Institute Laboratory Animal Welfare and Ethics Committee.

### Behavioral tests

**Fig. 1:**
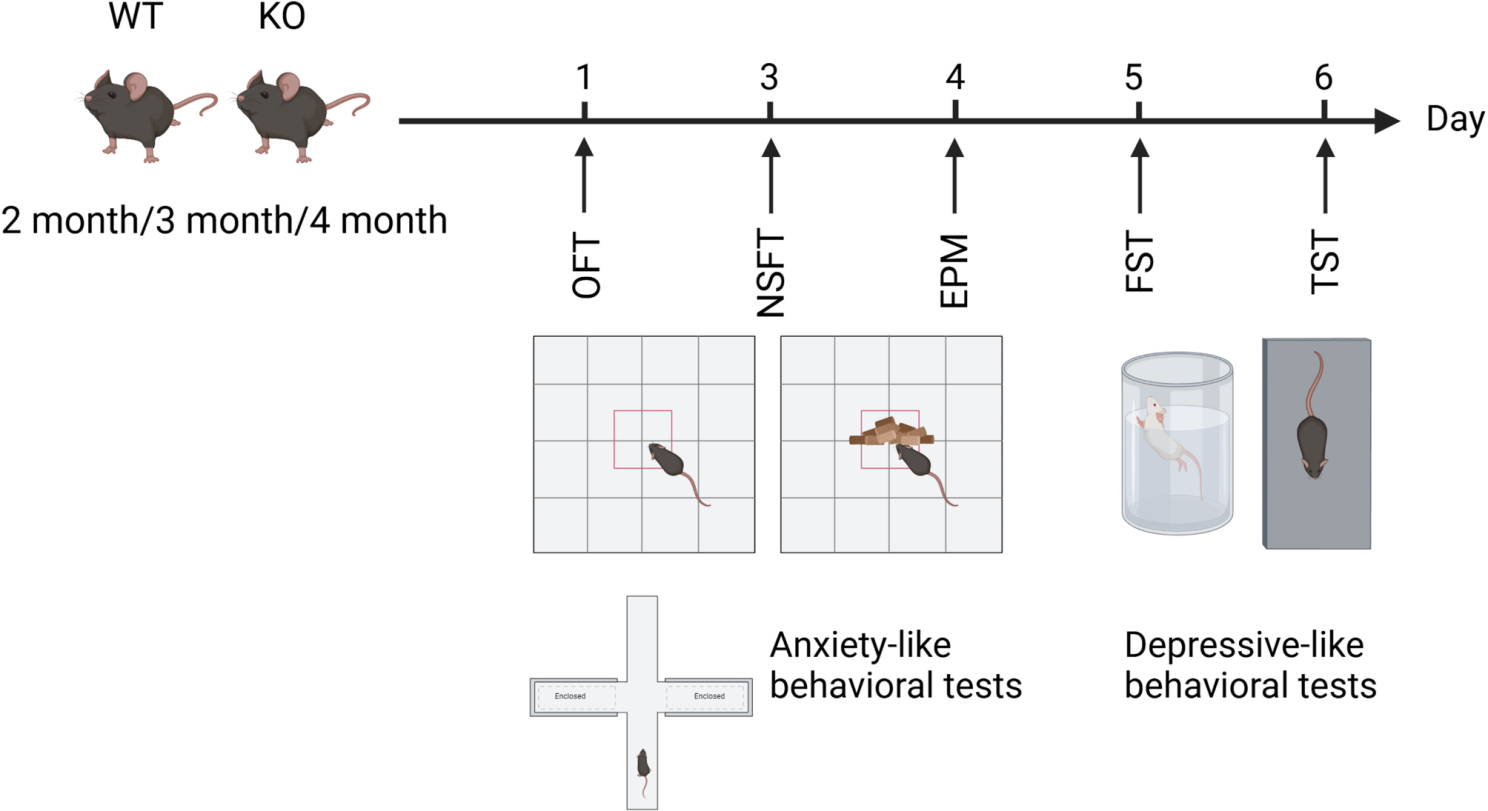
Flowchart of the emotional behavioral tests. Created with BioRender.com

To examine the emotional behaviors, a cohort of WT and Asm KO mice, ranging in age from 2 to 4 months, were subjected to a series of behavioral tests as illustrated in Fig. 1. All behavioral tests began 30 min after the mice had habituated to the test environment. And after conducting each mouse behavioral test, the testing area was carefully cleaned with 75% alcohol to eliminate any remaining odors. The behavioral tests were performed during the light cycle between 09:00 to 16:00 by an investigator blind to the genotype of the tested animals.

### Anxiety-like behavioral tests

#### Open field test (OFT)

Each single WT and KO mouse was sequentially placed into a cubic arena (50 × 50 × 50 cm) for 10 min, oriented towards the same wall, and allowed to explore the arena freely. Within this space, a virtual square of equal distance from the periphery (30 × 30 cm) was defined as the “central area”. Video recordings were captured and subsequently analyzed using EthoVision XT software (Noldus, Netherlands). The total distance, time spent in the center area, rearing times, grooming times were assessed. Besides, at the end of each session, the fecal pellets were collected and counted for assessing fecal output.

#### Novelty-suppressed feeding test (NSFT)

Mice were deprived from food for 24 h before the test. Subsequent to the fasting period, each mouse was placed in the corner of a square arena (50 × 50 × 50 cm), facing the corner, with a piece of food positioned in the center. The latency (in seconds) before a mouse initiated eating within 5 min after being placed in the arena was recorded.

#### Elevated plus maze test (EPM)

The device is composed of two open arms (30 cm×5 cm) and two closed arms (30 cm×5 cm×15 cm) intersecting vertically, with a square area of 5 cm×5 cm in the middle, elevated 40 cm above the ground. Each mouse was individually positioned in the 5 cm×5 cm central square, facing toward one of the open arms, and subsequently given a 5-minute period to freely explore the maze. The duration and frequency of their entries into the open and closed arms were determined and analyzed using EthoVision XT software (Noldus, Netherlands) as indicators for anxiety levels.

### Depression-like behavioral tests

#### Forced swimming test (FST)

Behavioral despair was evaluated using FST in a transparent glass cylinder with a height of 30 cm and a diameter of 12 cm. The cylinder was filled with water at a temperature ranging from 23 to 25 °C, reaching a depth of 18 cm to prevent mice from touching the bottom with their tails. Mice were placed in water and forced to swim for 6 min, and the cumulative immobility time was recorded for the last 4 min. The water was changed after each trial. Immobility time is characterized by the absence of active movements, which means the mice no longer struggled in the water, floated, or made only minor movements of their limbs to maintain their head afloat, except for those necessary for respiration.

#### Tail suspension test (TST)

Mice were suspended upside down approximately 30 cm above the floor by using adhesive tape positioned 1 cm from the tip of their tails. The immobility duration of the TST was assessed during the final 4 min of a 6-minute testing session. Mice were considered immobile only when no limb or body movements were observed, except those related to respiration.

### Cerebral blood flow (CBF) measurement

The mice were placed in a ventral recumbent position on a heating pad under deep anesthesia induced by tribromoethyl alcohol, with the rectal temperature maintained between 36.5 and 37.5 °C. The fur over the top of the skull was shaved, followed by a ∼ 1 cm incision along the sagittal suture. The skin was then retracted using sutures, and a thin layer of glycerin was applied to maintain skull moisture. Subsequently, CBF was monitored through the skull with a laser speckle blood flow imager (Moor Instruments, England & Wales). CBF of WT and KO mice was recorded at different month of ages.

### Brain tissue preparation

At the end of behavioral tests and CBF detection, mice were anesthetized via intraperitoneal injection of tribromoethanol (240 mg/kg) and then transcardially perfused with 20 ml PBS (pH 7.4) followed by 30 ml 4% paraformaldehyde. Brain samples were then rapidly removed and post-fixed in 4% paraformaldehyde for a minimum of 24 h, followed by dehydration in ethanol and embedding in paraffin. Coronal sections at the level of the prefrontal cortex were further processed for immunohistochemistry staining. For RNA-seq and q-PCR analyses, mice were transcardially perfused with 50 ml PBS, after which brain samples were rapidly removed and stored at -80 °C.

### RNA-seq

First, total RNA was isolated from the prefrontal cortex using a Total RNA Extractor (Sangon, China). Qubit 2.0 (Invitrogen, USA) was utilized for RNA concentration measurement, while agarose gel electrophoresis was employed to assess RNA integrity and detect genome contamination. The high-quality RNA samples were subsequently utilized for library preparation and sequencing. Libraries were generated using the VAHTSTM mRNA-seq V2 Library Prep Kit (Illumina, USA) according to the manufacturer’s recommendations. Qualified libraries were then sequenced using the Illumina Hiseq platform. FastQC was employed to assess the quality of the sequenced data. Raw reads were filtered by Trimmomatic to obtain high-quality clean reads for subsequent analysis. The clean reads were then aligned to the mouse reference genome using HISAT2. Gene expression levels of the transcripts were quantified by StringTie. The use of TPM (Transcripts Per Million) normalizes for gene lengths and sequencing discrepancies, allowing for direct comparison of gene expression across samples. DESeq2 was used to identify differentially expressed genes (DEGs) between WT and ASM KO mice. For gene sequencing, the Fold Change value and *q*-value (adjusted *p*-values following correction) were used as related indicators; normally, genes were deemed as significant differentially expressed if *q*-value ≤ 0.05 and log|2FoldChange|≥1.

### Bioinformatics analysis of DEGs

An analysis of Gene Ontology (GO) enrichment, Kyoto Encyclopedia of Genes and Genomes (KEGG) enrichment, and protein-protein interaction network (PPI) was performed to further investigate the differentially expressed genes. For gene function enrichment analysis, DEGs were annotated using the GO database (http://www.geneontology.org) to determine the count of genes in each term and to perform GO function statistics, and a hypergeometric test was then used to identify significantly enriched GO terms within the gene list compared to the background of the reference gene list. For pathway enrichment analysis, the KEGG database (https://www.kegg.jp/) was utilized to evaluate significantly pathway enriched in DEGs compared to a reference gene background, using the hypergeometric test. GO terms and KEGG pathways with *q*-value ≤ 0.05 were considered as significantly altered. A PPI network was established by integrating the DEGs into the STRING database (https://string-db.org/). Subsequent topological analysis of the network was performed using Cytoscape along with its NetworkAnalyzer function. Hub genes were then selected based on their topological properties in the entire network using the Degree algorithm in the cytoHubba plug-in.

### q-PCR

Total RNA was extracted from the prefrontal cortex using Total RNA Extractor (Sangon, China) according to the manufacturer’s instructions. RNA concentration and purity were detected by nucleic acid quantitative determination meter (Thermo, USA). The total RNA was reverse transcribed by using SweScript All-in-One RT SuperMix for qPCR (Servicebio, China). The primer sequences are detailed in Table 1, with GAPDH serving as the endogenous control. The relative gene expression was determined through Real-Time PCR system (Bio-Rad, USA) by using 2× Universal Blue SYBR Green qPCR Master Mix (Servicebio, China) according to the manufacturer’s recommended protocol.

**Table 1:** The primer sequences

Gene	Forward	Reverse
Tlr1	GAGGCATGAAGAGAGCGGAA	TAGGGGTGTCCACAATTGCC
Tlr2	GCCACCATTTCCACGGACT	GGCTTCCTCTTGGCCTGG
Ccl3	CCACTGCCCTTGCTGTTCTT	GCAAAGGGTGCTGGTTTCAA
Ccl4	TGTACCATGACACTCTGCAAC	CAACGATGAATTGGCGTGGAA
Ccl5	ACTATGGCTCGGACACCA	ACACACTTGGCGGTTCCT
Cxcl9	GTGGAGTTCGAGGAACCCTAG	ATTGGGGCTTGGGGCAAAC
Cd86	ACGGAGTCAATGAAGATTTCCT	GATTCGGCTTCTTGTGACATAC
Gadph	AGGTCGGTGTGAACGGATTTG	TGTAGACCATGTAGTTGAGGTCA

### Immunohistochemistry staining

After deparaffinization and hydration, the prefrontal cortex slices underwent antigen retrieval in 0.01 M citrate buffer (pH 6.0). Subsequently, the slices were treated with 3% H_2_O_2_ for 10 min, followed by blocking in 5% goat serum and overnight incubation with primary antibodies ionized calcium binding adaptor molecule 1 (iba-1, 1:500, Servicebio, China) and glial fibrillary acidic protein (GFAP, 1:500, Servicebio, China). This was followed by incubation with corresponding secondary antibodies and HRP for two periods of 30 min each. DAB (Vector, USA) was applied for 2 min. The sections were counterstained with hematoxylin to staining cell nucleus. After permeabilization, the tissue section was preserved by mounting solution.

### Statistical analysis

Data are expressed as mean ± SD. Differences between groups were assessed by the *t*-test using IBM SPSS Statistics 26.0 software, and *p* value ≤ 0.05 was considered statistically significant. For RNA-seq data analysis, *q* value (adjusted *p* values following correction) ≤ 0.05 was considered statistically significant.

## Results

### Increased anxiety-like behaviors in asm KO mice

**Fig. 2:**
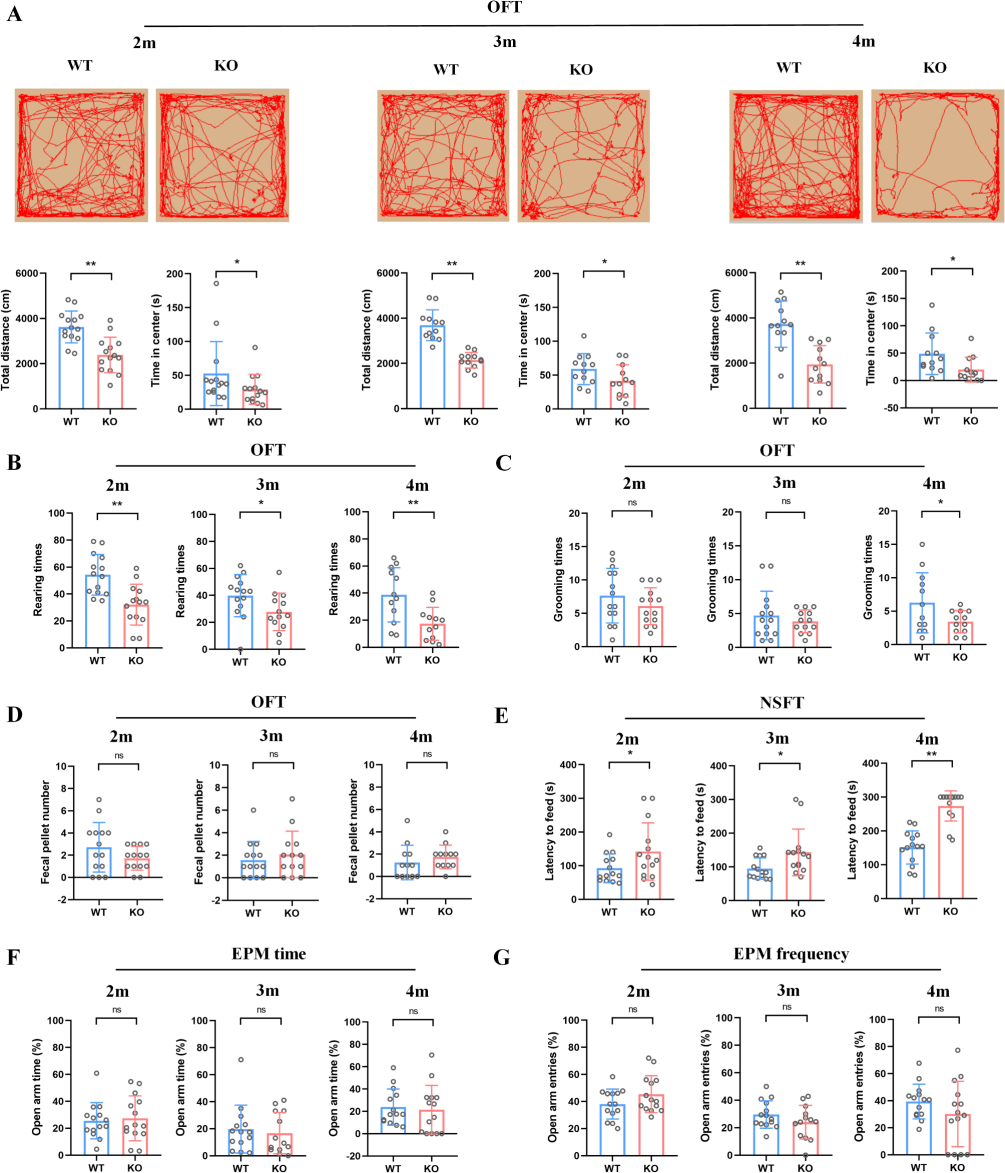
Anxiety-like behaviors in Asm KO and WT mice. (**A**) The representative images depicting the activity traces of mice at different ages in the OFT; the total distance (left) and the time spent in the center (right) of mice at different ages in the OFT. (**B**) The rearing times of mice at different ages in the OFT. (**C**) The grooming times of mice at different ages in the OFT. (**D**) The fecal pellet number of mice at different ages in the OFT. (**E**) The latency to feed of mice at different ages in the NSFT. (**F**) The open arm time of mice at different ages in the EPM. (**G**) The open arm entries of mice at different ages in the EPM. All data are shown as mean ± SD. * *p* < 0.05; ** *p* < 0.01; ns means no significance vs. WT mice. *n* = 12–14

To examine the potential involvement of the Asm in anxiety-like behavior, a series of anxiety behavioral experiments were conducted. In the open field test, as shown in Fig. 2A, Asm KO mice exhibited reduced activity traces and total distance during the 10-minute assessment (*p* = 0.0001, 2 m; *p* = 0.0000, 3 m; *p* = 0.0000, 4 m) compared to WT mice, as well as a decrease in cumulative times in the central area in OFT (*p* = 0.0454, 2 m; *p* = 0.0402, 3 m; *p* = 0.0180, 4 m). Moreover, the rearing times (Fig. 2B, *p* = 0.0003, 2 m; *p* = 0.0223, 3 m; *p* = 0.0026, 4 m) and grooming times (Fig. 2C, *p* = 0.1234, 2 m; *p* = 0.2124, 3 m; *p* = 0.0304, 4 m) were also decreased in KO mice, while no significant difference was observed in the fecal pellet number (Fig. 2D, *p* = 0.0737, 2 m; *p* = 0.2453, 3 m; *p* = 0.1829, 4 m) between the two genotypes. These findings collectively indicate a reduction in spontaneous activity and exploratory behavior, indicative of anxiety in Asm KO mice. In the novelty-suppressed feeding test, compared with the WT mice, KO mice showed a delayed latency to feed after 24 h of fasting (Fig. 2E, *p* = 0.0329, 2 m; *p* = 0.0143, 3 m; *p* = 0.0000, 4 m), and this delay increased with age. No genotype effects were observed on the ratio of open arm time (Fig. 2F, *p* = 0.3783, 2 m; *p* = 0.3180, 3 m; *p* = 0.3790, 4 m) and open arm entries (Fig. 2G, *p* = 0.0660, 2 m; *p* = 0.1025, 3 m; *p* = 0.1284, 4 m) in the elevated plus maze test. Taken together, these results suggest that Asm KO mice exhibit increased anxiety-like behaviors.

We next assessed the impact of Asm ablation on depressive-like behavior by using a forced swim test and tail suspension test. Asm KO mice displayed a significant decrease in immobility in the tail suspension test at 3 and 4 months of age (Fig. S1A, *p* = 0.0994, 2 m; *p* = 0.0002, 3 m; *p* = 0.0072, 4 m), with a noticeable difference observed solely in the forced swim test at 4 months of age (Fig. S1B, *p* = 0.1764, 2 m; *p* = 0.3655, 3 m; *p* = 0.0285, 4 m). Although Asm KO mice displayed reduced depressive-like behaviors compared to WT mice based on our findings, it was crucial to emphasize that WT mice did not serve as a kind of disease model for depression. Therefore, the deficiency in ASM did not indicate a reduction in depressive-like behaviors under pathological conditions but rather a partial amelioration of such behaviors under normal physiological states. No significant gender differences were observed in the behavioral tests, as shown in the Supplementary data (Fig. S2 and S1).

### Reduced cerebral blood flow in asm KO mice

**Fig. 3:**
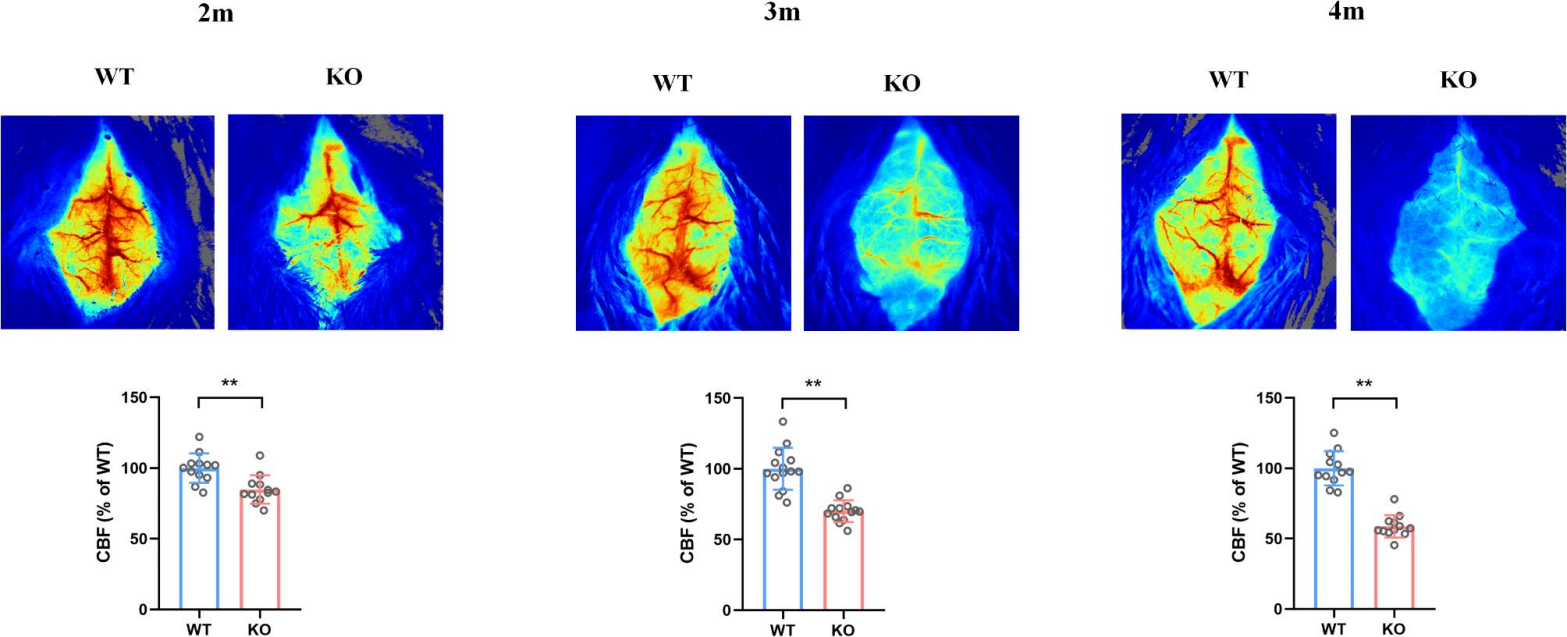
Cerebral blood flow in Asm KO and WT mice. The representative cortical CBF images (Top) and the quantitative analysis of CBF (Bottom) obtained from laser speckle blood flow imager in mice at different ages. All data are shown as mean ± SD. * *p* < 0.05; ** *p* < 0.01; ns means no significance vs. WT mice. *n* = 12–14

Research has indicated that alterations in cerebral blood flow are linked to anxiety. Therefore, we investigated the differences in CBF between WT and Asm KO mice at various ages. The result indicated a progressive decline in cerebral blood flow in KO mice with the increase of age compared to WT mice (Fig. 3 and Fig. S4, *p* = 0.0008, 2 m; *p* = 0.0000, 3 m; *p* = 0.0000, 4 m).

### Alterations in mRNA expression profiling in the prefrontal cortex following asm KO

**Fig. 4:**
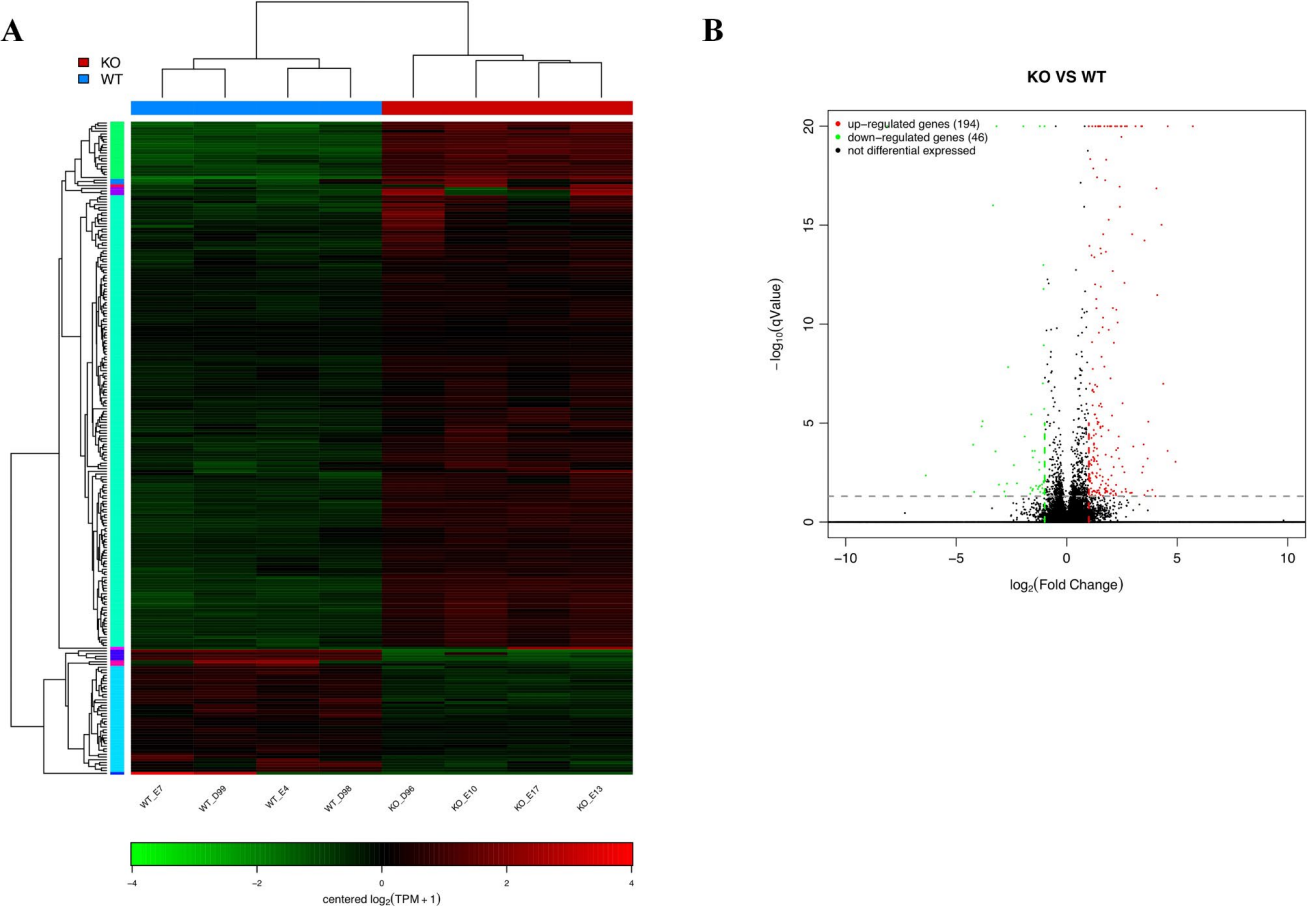
mRNA expression profiling in the prefrontal cortex of Asm KO and WT mice. (**A**) The heatmap of RNA-seq data showing the expression levels of DEGs in ASM KO and WT mice. Four biological replicates were performed for each genotype mice (repeat 1, 2, 3 and 4). The WT mice is denoted as WT. WT_E7, WT_D99, WT_E4, WT_D98 represent the WT mice. The ASM KO mice is denoted as KO. KO_D96, KO_E10, KO_E17, KO_E13 represent the ASM KO mice. (**B**) The volcano plot of differential expression in ASM KO and WT mice. Red dots show the genes that were significantly upregulated following ASM KO while green dots show genes that were significantly downregulated after ASM KO

To uncover the potential regulation mechanisms of anxiety like behaviors following Asm knockout, we conducted a transcriptomics analysis of the prefrontal cortex in WT and KO mice at 4 months of age. RNA-Seq was performed on a total of 8 samples from WT and KO mice. The overall gene expression distribution of each RNA-seq library is illustrated in Fig. 4A. A total of 240 DEGs were identified, with 194 genes showing up-regulation and 46 genes displaying down-regulation (*q* ≤ 0.05 and log|2FoldChange|≥1) as shown in Fig. 4B and Dataset S1. These results provide evidence at the transcriptional level that Asm knockout altered gene expression profiling in the prefrontal cortex, suggesting potential involvement of these DEGs in the development of anxiety.

### Identification of key genes associated with anxiety-like behaviors induced by asm KO

**Fig. 5:**
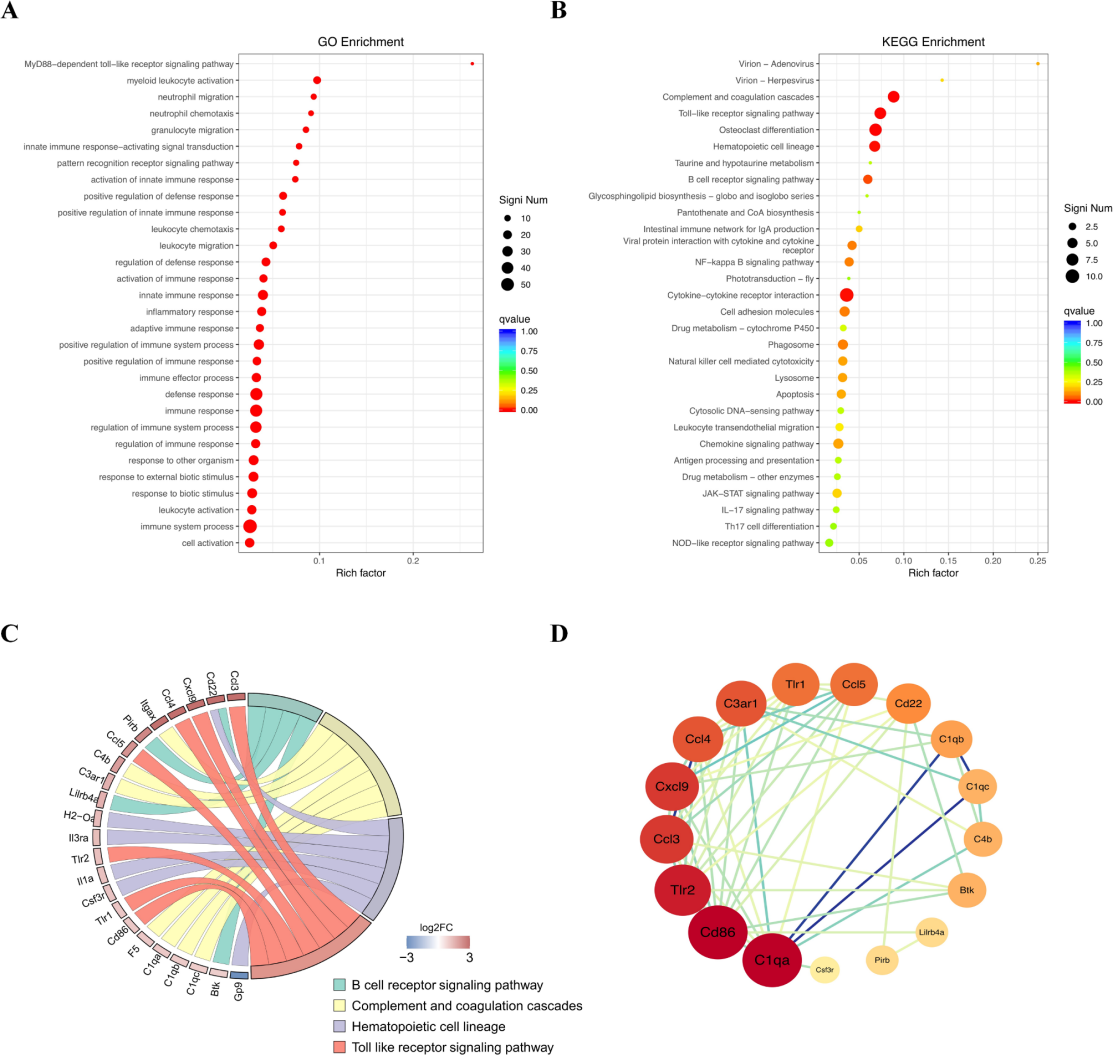
Analysis of key genes associated with anxiety-like behaviors induced by Asm KO. (**A**) The bubble chart of GO enrichment analysis of the DGEs (top 30 results). Rich factor refers to the DEGs annotated to a specific GO term, divided by the total number of genes annotated to that GO term. Meanwhile, Signi Num refer to the number of DEGs annotated to the corresponding GO term. (**B**) The bubble chart of KEGG pathway enrichment analysis DGEs (top 30 results). Rich factor refers to the DEGs annotated to a specific KEGG pathway, divided by the total number of genes annotated to that KEGG pathway. Meanwhile, Signi Num refer to the number of DEGs annotated to the corresponding KEGG pathway. (**C**) The chord diagram of the four pathways involved in the immune system section and their corresponding DEGs. (**D**) PPI analysis the 23 differentially expressed immune-related genes

The GO database categorizes gene functions into three components: Cellular Component (CC), Molecular Function (MF), and Biological Process (BP). We performed GO enrichment analysis on the 240 DEGs and found that they were enriched in 98 GO terms (*q* ≤ 0.05) of BP. Most of these terms were associated with the immune system (Fig. 5A), particularly immune system related process or response, as well as immune cell migration, such as immune system process, defense response, immune response, regulation of immune system process, leukocyte migration, granulocyte migration, and others. In addition, only one MF term, Toll-like receptor binding, was found to be significantly enriched (*q* = 0.0052), with no enrichment observed for any CC terms (Dataset S2). The clustering of all enriched BPs indicated that Asm KO had the most significant impact on immune system. Moreover, we conducted KEGG enrichment analysis on these 240 DEGs and observed significant enrichment in six KEGG pathways (*q* ≤ 0.05) (Dataset S3; Fig. 5B). Among these pathways, four were related to the immune system, including complement and coagulation cascades, Toll-like receptor signaling pathway, hematopoietic cell lineage, and B cell receptor signaling pathway, with a total of 23 corresponding annotated genes (Fig. 5C). These findings indicate that disruptions in immune system signaling might be correlated to anxiety-like behavior in mice following Asm KO. Immune signaling contributes to the regulation of neurobiological processes that impact emotional disorders such as anxiety. To further investigate the key immune-related genes involved in Asm KO-induced anxiety-like behavior, we constructed PPI networks for hub genes derived from the DEGs in KEGG significantly enriched immune system-related pathways. As shown in Fig. 5D, based on the Degree algorithm, genes like cluster of differentiation 86 (Cd86), complement C1q A chain (C1qa), toll-like receptor 2 (Tlr2) and others were found to be ranked higher, suggesting their pivotal roles in the immune signaling pathway network. Notably, we observed the genes involved in Toll-like receptor signaling pathway with higher rankings in the PPI network, including toll-like receptor 1 (Tlr1), Tlr2, C-C motif chemokine ligand 3/4/5 (Ccl3/4/5), C-X-C motif chemokine ligand 9 (Cxcl9), Cd86. Consequently, we hypothesize that the Toll-like receptor signaling pathway may serve as the underlying mechanism of Asm KO-induced anxiety-like behavior.

### Verification of the mRNA levels of key genes

**Fig. 6:**
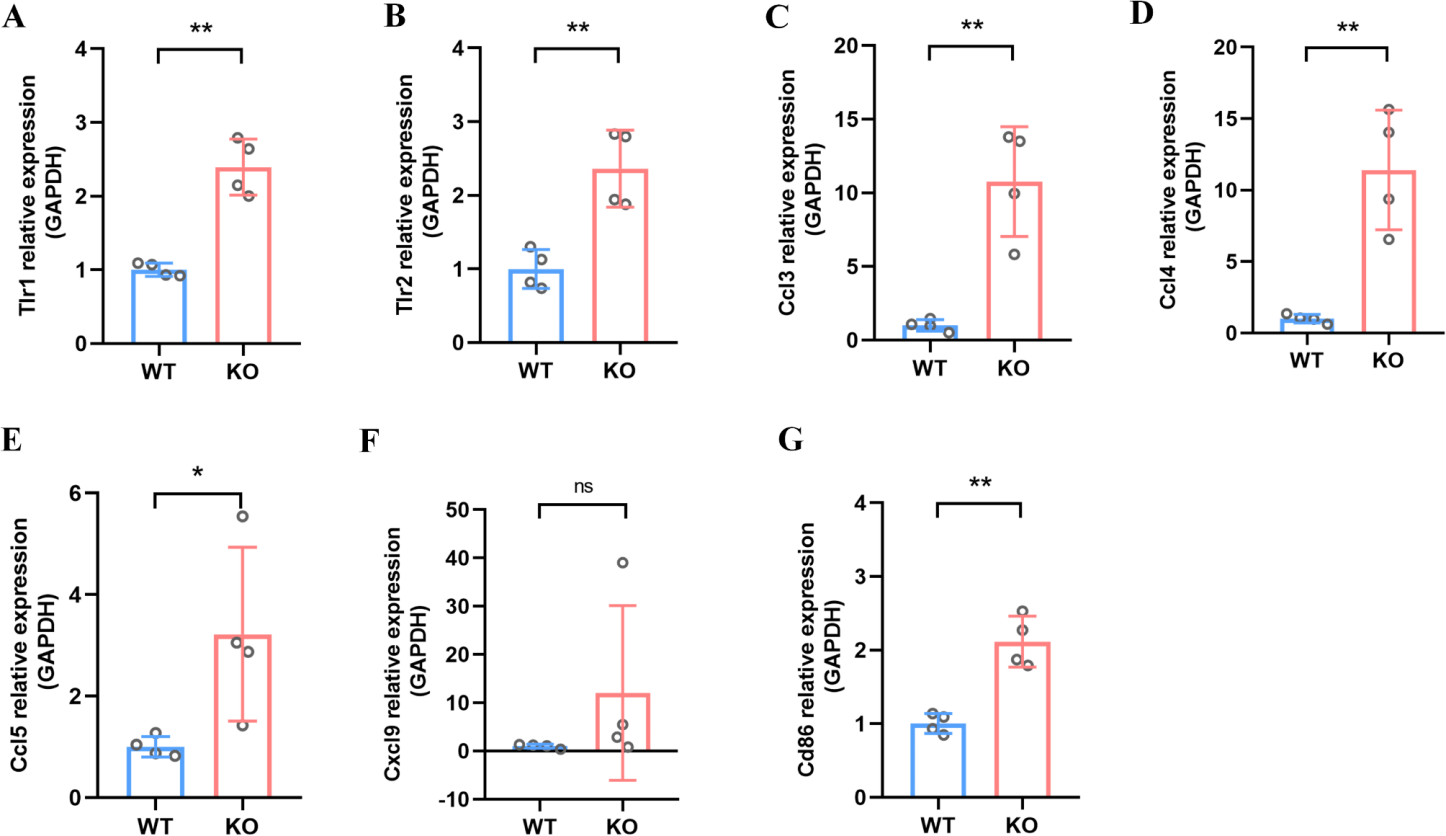
mRNA expression of immune-related genes in Toll-like receptor signaling pathway. (**A**) The mRNA expression of Tlr1 in WT and ASM KO mice. (**B**) The mRNA expression of Tlr2 in WT and ASM KO mice. (**C**) The mRNA expression of Ccl3 in WT and ASM KO mice. (**D**) The mRNA expression of Ccl4 in WT and ASM KO mice. (**E**) The mRNA expression of Ccl5 in WT and ASM KO mice. (**F**) The mRNA expression of Cxcl9 in WT and ASM KO mice. (**G**) The mRNA expression of Cd86 in WT and ASM KO mice. All data are shown as mean ± SD. * *p* < 0.05; ** *p* < 0.01; ns means no significance vs. WT mice. *n* = 4

To validate the RNA-seq data, seven genes enriched in the Toll-like receptor signaling pathway were be analyzed by q-PCR. As shown in Fig. 6A-G, compared with WT mice, the mRNA expression levels of Tlr1 (*p* = 0.0019), Tlr2 (*p* = 0.0038), Ccl3 (*p* = 0.0065), Ccl4 (*p* = 0.0077), Ccl5 (*p* = 0.0400) and Cd86 (*p* = 0.0021) were significantly increased in ASM KO mice. Additionally, the expression level of Cxcl9 was also elevated in Asm KO mice, although the difference did not reach statistical significance (*p* = 0.1549). The q-PCR results generally aligned with the RNA-seq findings outlined earlier, offering further evidence that Toll-like receptor signaling was indeed activated in Asm KO-induced anxiety-like behavior.

### Activated immune response and inflammation in asm KO mice

**Fig. 7:**
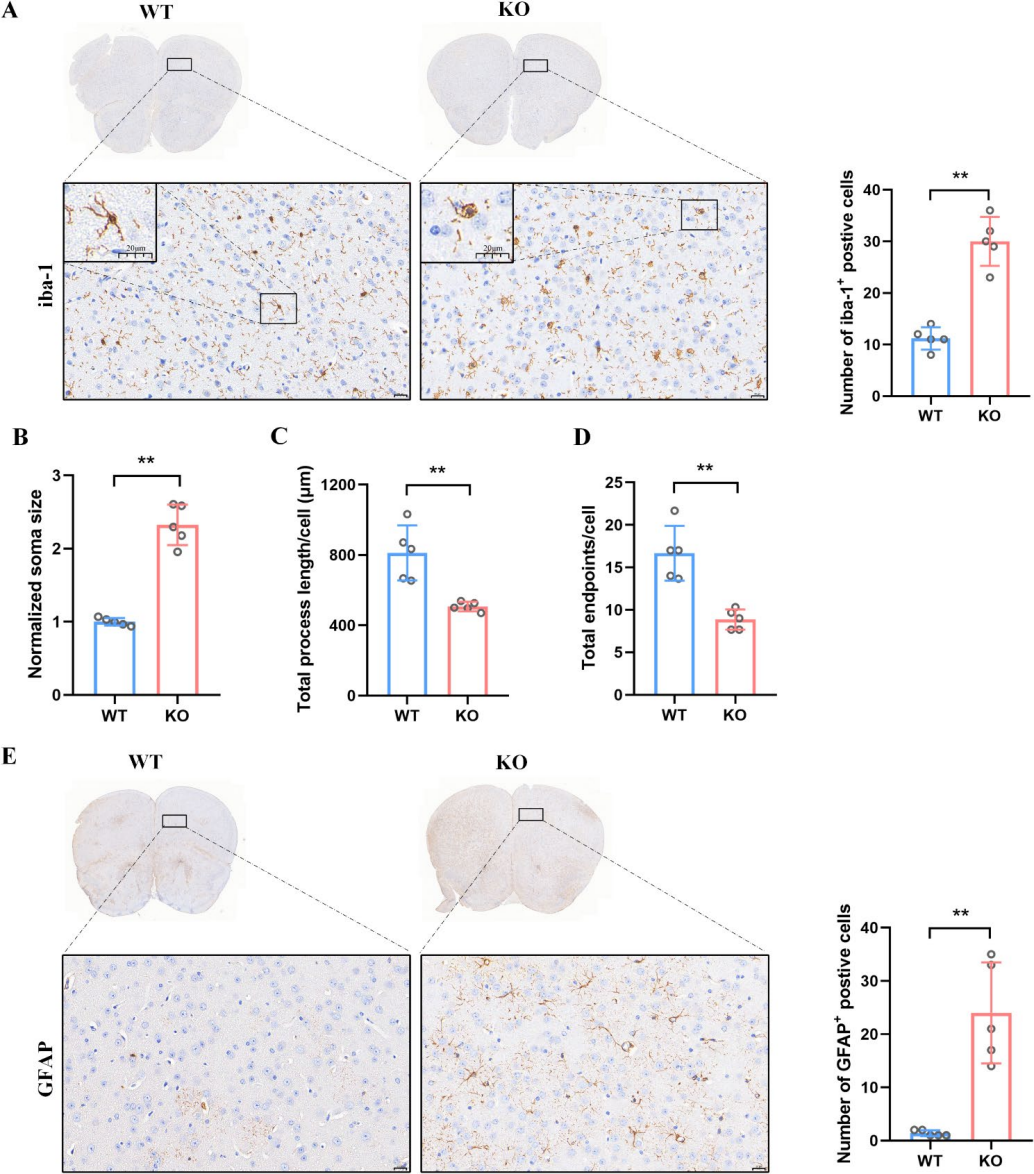
Immune cell and inflammation in Asm KO and WT mice. (**A**) The representative PFC immunohistochemical staining of iba-1 (left, Scale bar = 20 μm) and the summary of iba-1 immunohistochemical staining results (right). (**B**) The quantitative analysis of the soma size. (**C**) The quantitative analysis of the total process length. (**D**) The quantitative analysis of the total endpoints. (**E**) The representative PFC immunohistochemical staining of GFAP (left, Scale bar = 20 μm) and the summary of GFAP immunohistochemical staining results (right). All data are shown as mean ± SD. * *p* < 0.05; ** *p* < 0.01; ns means no significance vs. WT mice. *n* = 5

Toll-like receptor signaling is involved in activating innate and adaptive immune responses and plays a pivotal role in inflammation related diseases [15]. Macrophages constitute a significant group of immune cells involved in both innate and adaptive immune responses. Within the nervous system, microglia serve as a specialized subtype of macrophage. To verify the effect of Asm KO on the activation of immune response, iba-1, a specific marker of microglia, was detected. As shown in Fig. 7A, Asm KO mice exhibited markedly enhanced iba-1^+^ positive cells than WT mice (*p* = 0.0001). Interestingly, we also observed concurrent changes in the morphology of microglia in KO mice. Following Asm deficiency, microglia exhibited an increased soma size (Fig. 7B, *p* = 0.0002), but a decreased total process length (Fig. 7C, *p* = 0.0056) and endpoints (Fig. 7D, *p* = 0.0018), indicating microglial activation. Astrocytes can serve as immune support cells in the nervous system, being activated by microglia and collaborating in inflammatory responses. GFAP is commonly used as a marker for astrocyte reactivity and inflammation. Consistent with the result of microglia, the number of GFPA-positive astrocytes in KO mice also showed a significant increase (Fig. 7E, *p* = 0.0030). The findings collectively suggest that the anxiety-like behavior induced by Asm KO is associated with an activated immune response and inflammation.

## Discussion

Although abnormal ASM activity has been reported to be associated with several kinds of psychiatric disorders, its specific role in the pathological process of anxiety remains incompletely understood. In the present study, we investigated the impact of Asm on emotions using Asm KO mice. Through a series of behavioral tests including OFT, NSFT and EPM, we found that Asm KO mice clearly showed increased anxiety-like behavior as compared with the WT mice. Importantly, employing mRNA transcriptional profiling coupled with bioinformatics analysis, we identified the activation of the Tlr1/2 signaling pathway following Asm knockout. This finding was further confirmed by the observed upregulation of Tlr1, Tlr2, Ccl3, Ccl4, Ccl5 and Cd86 mRNA levels using q-PCR. Additionally, immunohistochemical analysis revealed an increase in the marker of microglia iba-1 and the marker of astrocyte GFAP expression. These results collectively suggest an association between Asm-induced anxiety and neuroinflammation.

In this study, we performed several classical animal behavioral tests, including the OFT, NSFT and EPM, to evaluate the anxiety-like behavior in both WT and Asm KO mice. These tests are widely recognized for their high reliability and validity in evaluating anxiety levels in rodents [16]. The findings indicated that a deficiency in Asm resulted in anxiety-like behavior in mice in OFT and NSFT, but not in EPM. Specifically, Asm KO mice exhibited decreased exploration of a novel environment and spent less time in the center of the OFT. Additionally, they exhibited an increased latency to feed in the NSFT. However, in the EPM, there were no obvious differences in the percentage of open arm time or entries between WT and Asm KO mice, thus the results of EPM showed less evidence for Asm KO-induced anxiety-like behavior. Overall, the OFT and EPM are designed based on rodents’ innate aversion to open spaces, utilizing the conflict between their natural instinct for exploration and this aversion [17], providing an analogy to human anxiety symptoms. The behavioral profiles elicited in these tests encompass elements of neophobia, exploration, and approach/avoidance conflict. The NSFT is built upon the OFT by introducing the ‘Hunger Factor’, thereby creating a conflict in animals between their urge to feed and their reluctance to venture into open spaces [18]. In our tests, Asm KO mice appeared more fearful of the open field but did not exhibit fear of elevated open arms. The discrepancies in the outcomes of behavioral tests within the same study are not coincidental. Previous research has revealed a primary reason for such variations: little correlation among anxiety-related behaviors measured in different tests [19, 20]. Additionally, emotionality is a multi-dimensional parameter that can be examined from various perspectives. Consequently, environmental factors such as open spaces, lighting conditions, or elevated areas may evoke diverse behavioral responses [21]. Therefore, despite the EPM did not providing sufficient results, the combined results from the OFT and NSFT support the idea that Asm KO caused mice to enter an anxious emotional state.

Furthermore, our study revealed a significant decrease in CBF in Asm KO mice compared to WT mice. There seems to be a relationship between anxiety levels and cerebral blood flow. It was found in clinical study that subjects with high state anxiety exhibited lower CBF [22]. The CBF of the right insula/Heschl’s cortex decreased in patients with pulmonary nodules in an anxiety state [23]. Additionally, individuals suffering from ischemic stroke were more susceptible to developing anxiety disorders [24]. The CBF was significantly decreased in Omentin-1 knockout mice, which were susceptibility to anxiety-like behaviors [25]. In rats with hemorrhagic shock reperfusion, CBF in the bilateral hippocampus CA1 area showed a positive correlation with spatial exploration ability in the OFT [26]. The nervous system impairment caused by ischemia involves multiple pathways, including apoptosis, necroptosis, autophagy, pyroptosis and ferroptosis, which are associated with glutamate excitotoxicity, inflammation, and oxidative stress [27]. During cerebral ischemia, the elevated levels of extracellular glutamate initiated a positive feedback loop, whereby the activation of glutamate receptors further diminished ionic gradients and depleted ATP, both of which facilitated subsequent release of glutamate. While the hyperactivated glutamate system is considered one of the pathological mechanisms underlying anxiety disorders. Furthermore, ischemia can induce activation of resident microglia and astrocytes, as well as invasion of peripheral immune cells. These cells release cytokines and chemokines, such as TNF-α, IL-1β, IFN-γ, IL-6, iNOS, etc., which ultimately lead to neuronal death and trigger anxiety [28]. At present, the specific mechanisms through which ASM deficiency influences cerebral blood flow remain unknown, but certain studies might have provided some clues into this issue. Vascular smooth muscle cells (VSMCs) constitute one of the crucial cells governing the contraction and relaxation of vascular. A study revealed that Asm-deficient VSMCs displayed a distinct contractile phenotype compared to WT VSMCs [29], which might account for the reduced CBF in Asm-deficient mice. More experiments are needed to further investigate the specific mechanism by which Asm affects VSMCs phenotype and its relationship with the regulation of CBF.

Mice expose to an inescapable aversive situation will, following periods of agitation, cease attempting to escape and display an emotion similar to human despair. FST and TST are two commonly used tests for depression-like behavior [30], both of which are based on that principle. Asm KO mice exhibited a significant reduction in the duration of immobility when placed in a water-filled cylinder or suspended upside down by their tails. Our results revealed that mice lacking Asm showed decreased depressive-like behavior. It is imperative to note that this reduction did not manifest in a pathological context. This observation arose from the fact that the WT mice used for comparison were in a state of normal physiological state, rather than being in a depressive state. Clinically, elevated ASM activity was observed in patients with depression [12]. Antidepressants, such as the tricyclic antidepressant amitriptyline and the serotonin reuptake inhibitor fluoxetine, are known functional ASM inhibitors commonly prescribed for depressive disorder treatment [31]. Taken together, these findings suggested a potential association between high ASM expression and depression, supported by our observation of a partial amelioration of depression-like behaviors under normal physiological states induced by Asm deficiency.

The prefrontal cortex (PFC), a critical cortical region, plays a pivotal role in integrating and processing information to regulate negative emotional stimuli, such as anxiety. Consistent aberrations in PFC activity were observed in individuals with anxiety disorders [32]. To investigate the potential regulatory mechanisms underlying anxiety-like behaviors induced by Asm KO, we conducted a transcriptomic analysis on the PFC in both WT and KO mice. The findings revealed that the absence of Asm had a significant impact on the transcriptional profile in the PFC of mice, leading to the identification of 240 DEGs, including 194 significantly up-regulated genes and 46 significantly down-regulated genes. The depletion of Asm gene resulted in a significantly higher number of up-regulated genes compared to down-regulated genes, indicating that reduced Asm expression may lead to the activation of a larger set of genes. Some of the up-regulated DEGs with a higher fold change have been found to be associated with the immune system, such as lymphocyte antigen 9 (Ly9), Ccl3, Cd22 and Cxcl9. Additionally, other up-regulated DEGs, like C-type lectin domain family 7 member A (Clec7a) and lysozyme 2 (Lyz2), have been reported to have detrimental effects on other neurological disorders. A recent study demonstrated that Clec7a could exacerbate microglia-mediated synapse elimination, leading to the impairment of neurological function in ischemic stroke [33]. In a murine model of neurodegenerative disease, the in vivo expression level of Lyz2 exhibited an increase concomitant with disease progression [34]. These up-regulated genes might have been be implicated in the pathogenesis of anxiety disorders, necessitating further investigation to substantiate this association.

Moreover, we conducted GO functional enrichment analysis and KEGG pathway enrichment analysis of the DEGs to further explore the potential molecular mechanism underlying Asm KO-induced anxiety. Interestingly, all DEGs, except for one GO term of MF named Toll-like receptor binding, were enriched in 98 GO terms of BP, with the majority being associated with immune system, such as immune system process, defense response and immune response, etc. Similarly, with the exception of the osteoclast differentiation pathway and cytokine-cytokine receptor interaction pathway, four out of the six significantly enriched KEGG pathways were found to be associated with the immune system. These included complement and coagulation cascades, Toll-like receptor signaling pathway, hematopoietic cell lineage, and B cell receptor signaling pathway. These analyses once again demonstrated that alterations in the immune system might represent a potential mechanism for anxiety-like behavior mediated by Asm KO. The concept of neuroimmunology was established by Han Selye in the 1950s, marking the inception of research into the intricate interplay between the immune system and the nervous system [35]. Recent evidence increasingly supports the notion that neuroimmune system activation can influence changes in behavior. Accordingly, we conducted a PPI network analysis on 23 DEGs significantly enriched in immune system-related pathways in KEGG to further explore the key immune-related genes involved in Asm KO-induced anxiety-like behaviors. Through the PPI analysis, we discerned the crucial involvement of DEGs (Tlr1, Tlr2, Ccl3, Ccl4, Ccl5, Cxcl9, Cd86) that were significantly enriched in the Toll-like receptor signaling pathway. Furthermore, the expression of these key genes was confirmed through q-PCR. All of these results indicate that the Toll-like receptor signaling pathway could be the underlying mechanism of Asm KO-induced anxiety-like behavior.

Recent research has uncovered the intricate correlation between anxiety disorders and the immune system. Experimental evidences had demonstrated that the activation and dysregulation of immune cells and following inflammation could impact the neurotransmitter system, thereby influencing emotions and states of anxiety [36]. Toll-like receptor signaling pathway is intricately linked to the regulation of immune responses and inflammation. Toll-like receptors (Tlrs) are membrane-bound receptors predominantly expressed on innate immune cells. As the initiating molecules in signaling cascades, Tlrs promptly trigger innate immune responses by stimulating the synthesis of pro-inflammatory cytokines and enhancing the expression of costimulatory molecules upon recognition of pathogens. Tlr1 formed heterodimers with Tlr2 and became activated in response to neuropathogenesis [37]. Female offspring of mice fed with 2.5 times the recommended amount of folic acid exhibited increased anxiety behaviors, accompanied by an increase in Tlr1 mRNA expression levels in their brains [38]. Chronic stress is recognized as a severe risk factor leading to anxiety. Increased Tlr2 mRNA expression level was observed in a rat model of chronic unpredictable stress induced anxiety, which was reduced by the administration of the anxiolytic oregano extract [39]. Treatment with Tlr2 agonist lipoteichoic acid was found to induce anxiety-related behaviors in mice [40]. Furthermore, ethanol-withdrawal induced anxiogenic-like effects in WT mice, but not in Tlr2 KO mice [41]. Thus, Tlr1/2 could be the crucial inflammatory mediators in anxiety disorder. In our study, the increased expression levels of Tlr1/2 were also observed in Asm KO mice, indicating an association between activated Tlr1/2 and the anxiety-like behavior in Asm KO mice. Currently, there is a lack of direct evidence regarding the regulatory mechanism of Tlr1/2 expression by Asm. It is hypothesized that this may be associated with lipotoxicity resulting from sphingolipid accumulation. A study had shown that SM16:0 led to increased lysosomal membrane permeability, leading to efflux and subsequent induction of oxidative stress and neuronal death [42]. The damaged cell can activate Tlr1/2 by releasing damage-associated molecular patterns.

Chemokines and inflammatory mediators act as effector molecules in the downstream of the Toll-like receptor signaling pathway, playing a crucial role in recruiting immune cells to engage in inflammatory responses. Our findings revealed significant upregulation of chemokines Ccl3, Ccl4, Ccl5 and the inflammatory mediator Cd86 in Asm KO-induced anxiety. Tay-Sachs disease (TSD) is a fatal inherited lysosomal storage disorder. The early onset TSD mouse model Hexa−/−Neu3−/− mice demonstrated a high level of anxiety, along with elevated levels of Ccl2, Ccl3, Ccl4, Cxcl10 [43]. In mice with Autism spectrum disorder, gut microbiota transplantation treatment significantly ameliorated anxiety-like and repetitive behaviors and reduced levels of chemokines including Ccl3 and Ccl5 [44]. Acute restraint stress induced anxiety-like behavior in rats, accompanied by a significant upregulation of Ccl5. Similarly, stressed human participants also displayed elevated levels of Ccl5 [45]. All of these results further confirm our findings that dysregulation of chemokine expression is associated with anxiety. There was limited report on the direct correlation between Asm and these chemokines, and one of the studies in question appeared to yield conflicting results. They found Asm was essential for the production of Ccl5, and overexpression of Asm could lead to an up-regulation of Ccl5 [46]. This difference could be attributed to varying pathological conditions. The upregulation of chemokines, such as Ccl5, observed in our study was primarily associated with the downstream impacts of Tlr1/2 activation, which may be triggered by lipotoxicity resulting from Asm deficiency.

To further validated the downstream impacts of Tlr1/2 activation on immune cells in Asm KO induced anxiety, we assessed the expression of iba-1, a marker of microglial activation, and observed an increased number of microglia with the activated morphology. Besides, the increased Cd86 level also indicated Asm KO induce microglia towards an activated state. Activated microglia can induce astrocyte activation, thereby exacerbating neuroinflammation. Through GFAP immunohistochemical staining, we observed astrocyte activation in Asm KO mice. Taken together, these findings suggest that neuroinflammation may play a crucial role in the development of anxiety-like behavior in Asm KO mice.

As two of the most common psychiatric disorders, anxiety and depression usually occur simultaneously. About 40% of patients with depression were also diagnosed with anxiety disorders [47]. Additionally, several studies indicated a positive association between the upregulation of neuroinflammation and depression [48–50]. Interestingly, in our investigation, the upregulation of neuroinflammation triggered by Asm deficiency elicited changes in anxiety-like behaviors without a concomitant increase in depression-like behaviors; instead, a decrease was observed. This reduction, as previously mentioned, did not hold pathological significance, as the WT mice used as controls did not represent a type of disease model of depressive disorder. The intricate interplay among various neurobiological pathways implicated in anxiety and depression remains incompletely understood. It is plausible that compensatory mechanisms or the activation of alternative pathways in Asm KO mice may counterbalance the depression associated with typical neuroinflammatory response, thereby resulting in a distinct behavioral outcome. A study demonstrated an increase in p38-Kinase (p38K)-mediated neurogenesis in the hippocampus of Asm-deficient mice [51], indicating a potential alternative pathway to mitigate the depression linked with neuroinflammatory response. This finding might have elucidated the relatively lower level of depression observed in Asm KO mice despite the activation of a neuroinflammatory response compared to the normal physiological state seen in WT mice.

This study had some limitations. First, we utilized mice with systemic Asm deficiency and were unable to discern the impact of specific cell types such as neurons or microglia, deficient in Asm, on anxiety. Therefore, conditional knockout mice with different cell types need to be established for further research. Second, we did not further investigate the mechanism by how Asm upregulates Tlr1/2 expression, and whether there is a direct protein interaction with Tlr1/2 or indirect activation of Tlr1/2 through other pathways such as accumulated sphingomyelin needs to be verified by additional experiments.

In conclusion, our findings demonstrated that Asm KO mice exhibited emotional alterations characterized by enhanced anxiety-like behavior. This effect might have been achieved through modulating neuroinflammation by activating microglial and astrocyte. The underlying molecular mechanism was attributed to the activation of Tlr1/2 signaling pathway and subsequent induction of chemokines and inflammatory mediators. These results suggested that Asm represents a potential novel target for the treatment of anxiety disorders.

## Electronic supplementary material

Below is the link to the electronic supplementary material.

**Supplementary Material 1: Fig. S1** Depression-like behaviors in Asm KO and WT mice. **Fig. S2** Anxiety-like behaviors in female and male Asm KO and WT mice. **Fig. S3** Depression-like behaviors in female and male Asm KO and WT mice. **Fig. S4** Cerebral blood flow in female and male Asm KO and WT mice

**Supplementary Material 2: Dataset S1** Up and down regulation of DEGs identified by Asm-knockout

**Supplementary Material 3: Dataset S2** GO enrichment results of DEGs

**Supplementary Material 4: Dataset S3** KEGG enrichment results of DEGs

## Data Availability

The datasets used and/or analyzed during the current study are available from the corresponding author on reasonable request.

## Abbreviations

Asm/ASM: Acid sphingomyelinase
BP: Biological process
C1qa: Complement C1q A chain
CBF: Cerebral blood flow
CC: Cellular component
Ccl3/4/5: C-C motif chemokine ligand 3/4/5
Cd86: Cluster of differentiation 86
Clec7a: C-type lectin domain family 7 member A
Cxcl9: C-X-C motif chemokine ligand 9
DEGs: Differentially expressed genes
EPM: Elevated plus maze test
FST: Forced swimming test
GFAP: Glial fibrillary acidic protein
GO: Gene Ontology
iba-1: Ionized calcium binding adaptor molecule 1
KEGG: Kyoto Encyclopedia of Genes and Genomes
KO: Knockout
Ly9: Lymphocyte antigen 9
Lyz2: Lysozyme 2
MF: Molecular function
NSFT: Novelty-suppressed feeding test
OFT: Open field test
p38K: P38-Kinase
PFC: Prefrontal cortex
PPI: Protein-protein interaction network
SM: Sphingomyelin
Tlr1: Toll-like receptor 1
Tlr2: Toll-like receptor 2
Tlrs: Toll-like receptors
TPM: Transcripts per million
TSD: Tay-Sachs disease
TST: Tail suspension test
VSMCs: Vascular smooth muscle cells
WT: Wild-type
