# Supplementary material for: Acid sphingomyelinase modulates anxiety-like behavior likely through toll-like receptor signaling pathway
Source: Mol Brain. 2025 Feb 4;18:8. doi: 10.1186/s13041-025-01178-x (PMC11796198; doi:10.1186/s13041-025-01178-x)
Supplement: Supplementary file 1 — Supplementary Material 1: Fig. S1 Depression-like behaviors in Asm KO and WT mice. Fig. S2 Anxiety-like behaviors in female and male Asm KO and WT mice. Fig. S3 Depression-like behaviors in female and male Asm KO and WT mice. Fig. S4 Cerebral blood flow in female and male Asm KO and WT mice [file 13041_2025_1178_MOESM1_ESM.docx]

**
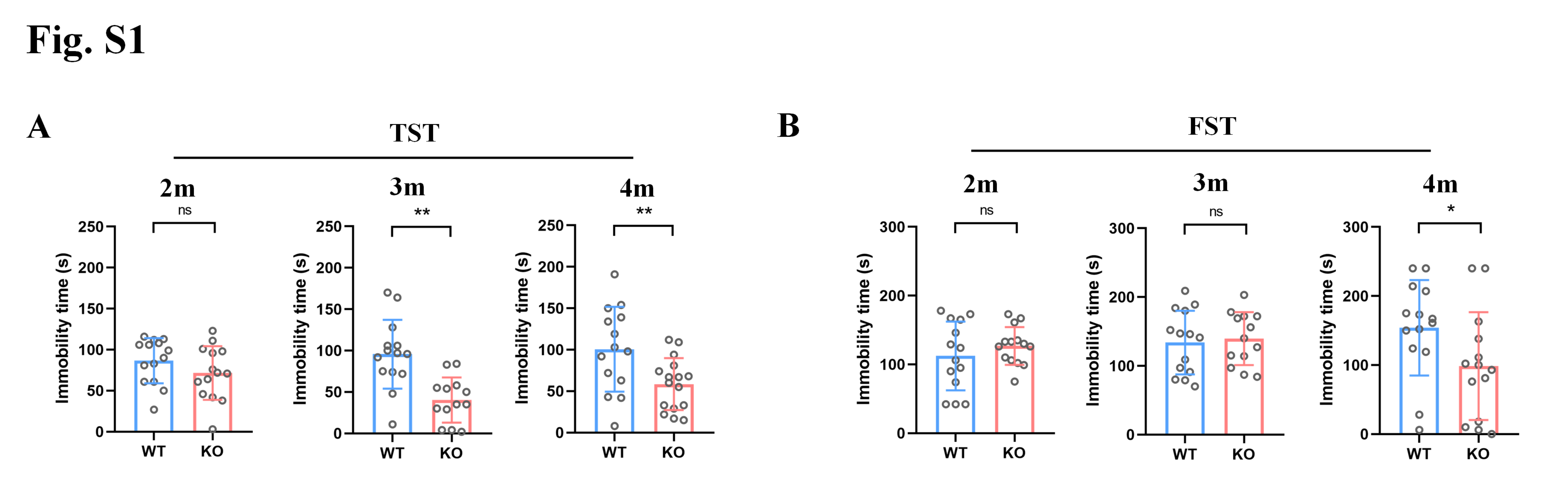
**

**Fig. S1 Depression-like behaviors in Asm KO and WT mice. A.** The immobility time of mice at different ages in the TST. **B.** The immobility time of mice at different ages in the FST. All data are shown as mean±SD. * *p* < 0.05; ** *p* < 0.01; ns means no significance vs WT mice. *n*=13-16.

**
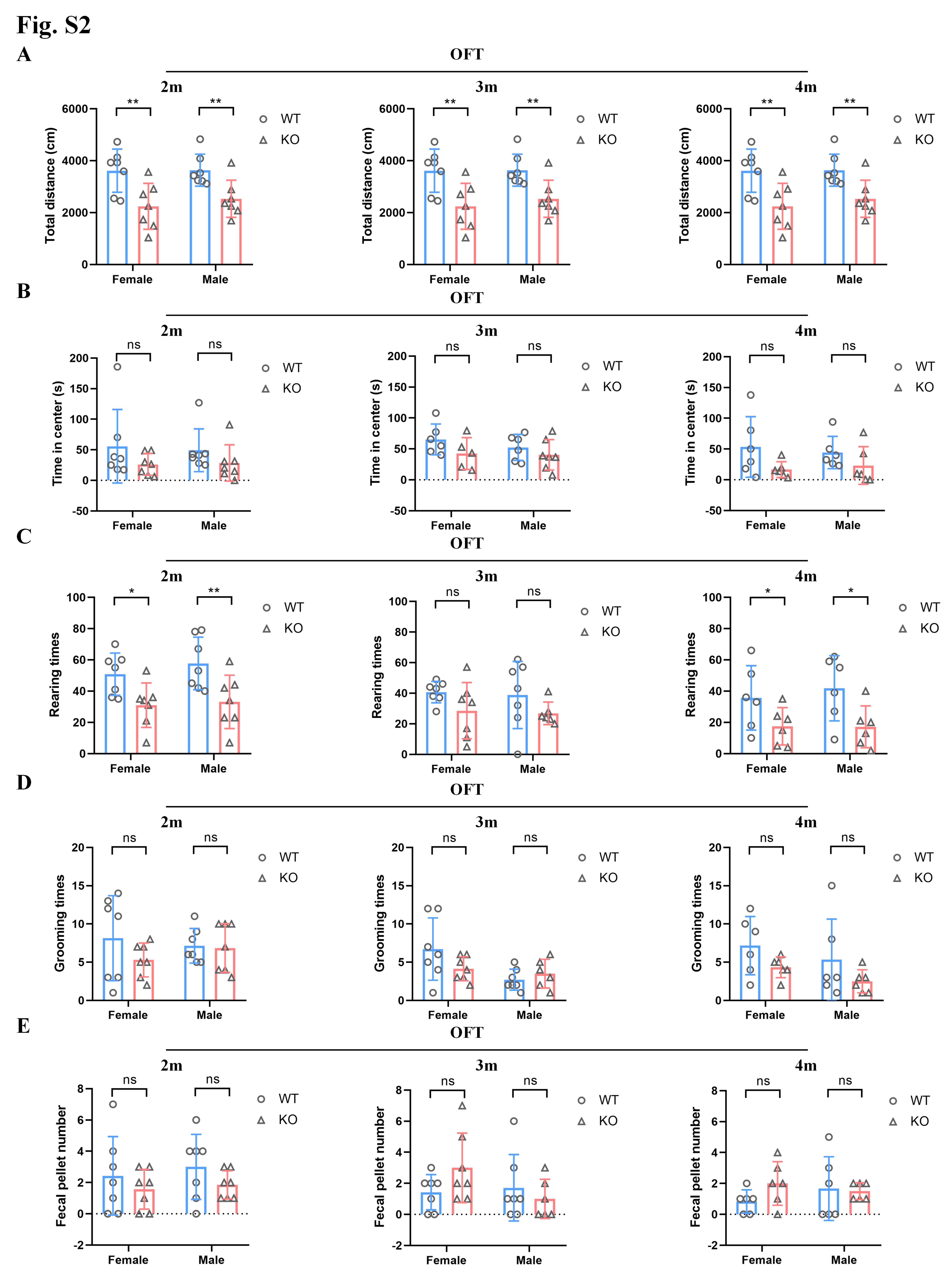
**

**
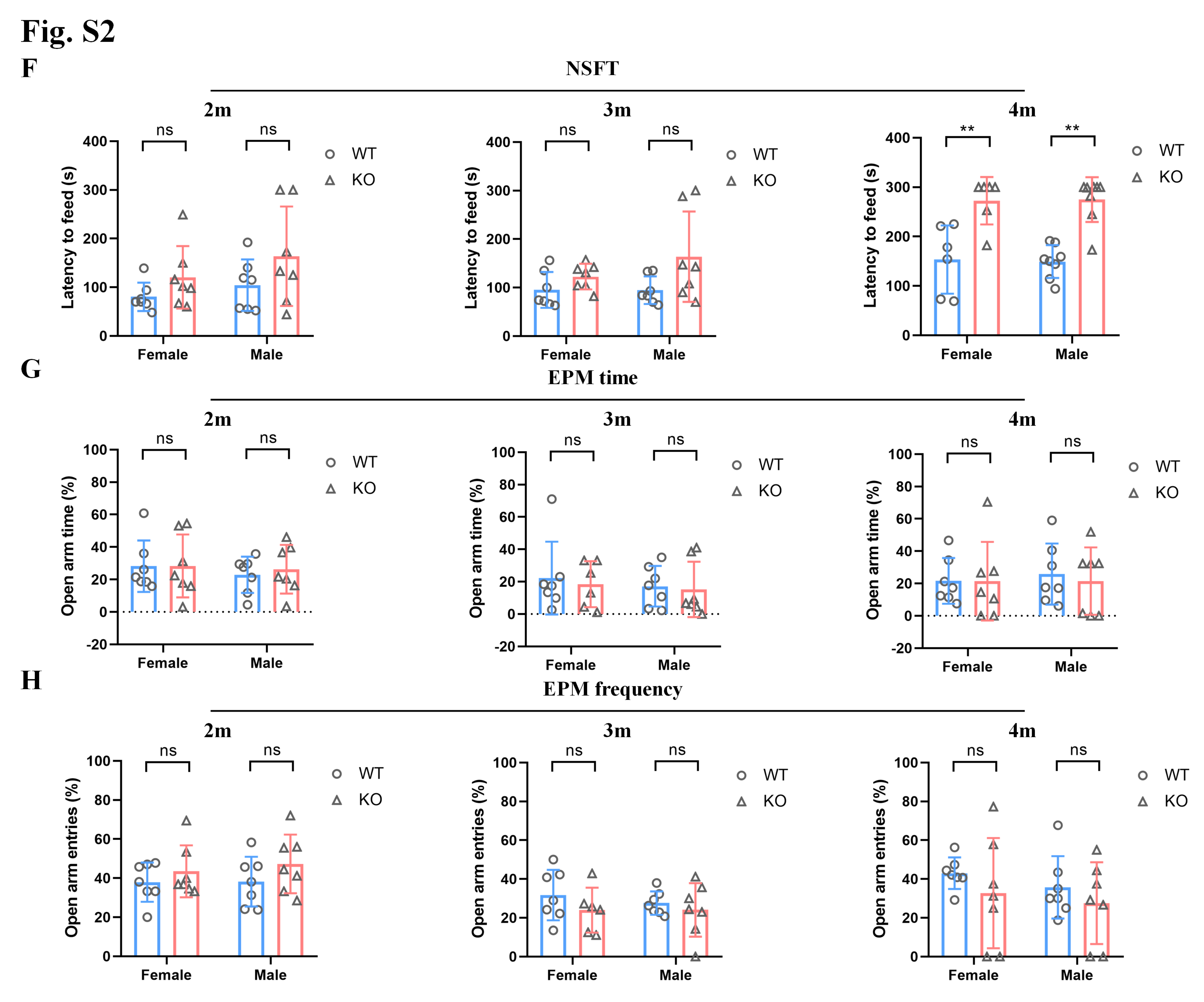
**

**Fig. S2 Anxiety-like behaviors in female and male Asm KO and WT mice. A.** The total distance at different ages in the OFT. **B.** The time spent in the center of mice at different ages in the OFT. **C.** The rearing times of mice at different ages in the OFT. **D.** The grooming times of mice at different ages in the OFT. **E.** The fecal pellet number of mice at different ages in the OFT. **F.** The latency to feed of mice at different ages in the NSFT. **G.** The open arm time of mice at different ages in the EPM. **H.** The open arm entries of mice at different ages in the EPM. All data are shown as mean±SD. * *p* < 0.05; ** *p* < 0.01; ns means no significance vs WT mice. *n*=5-8.

**
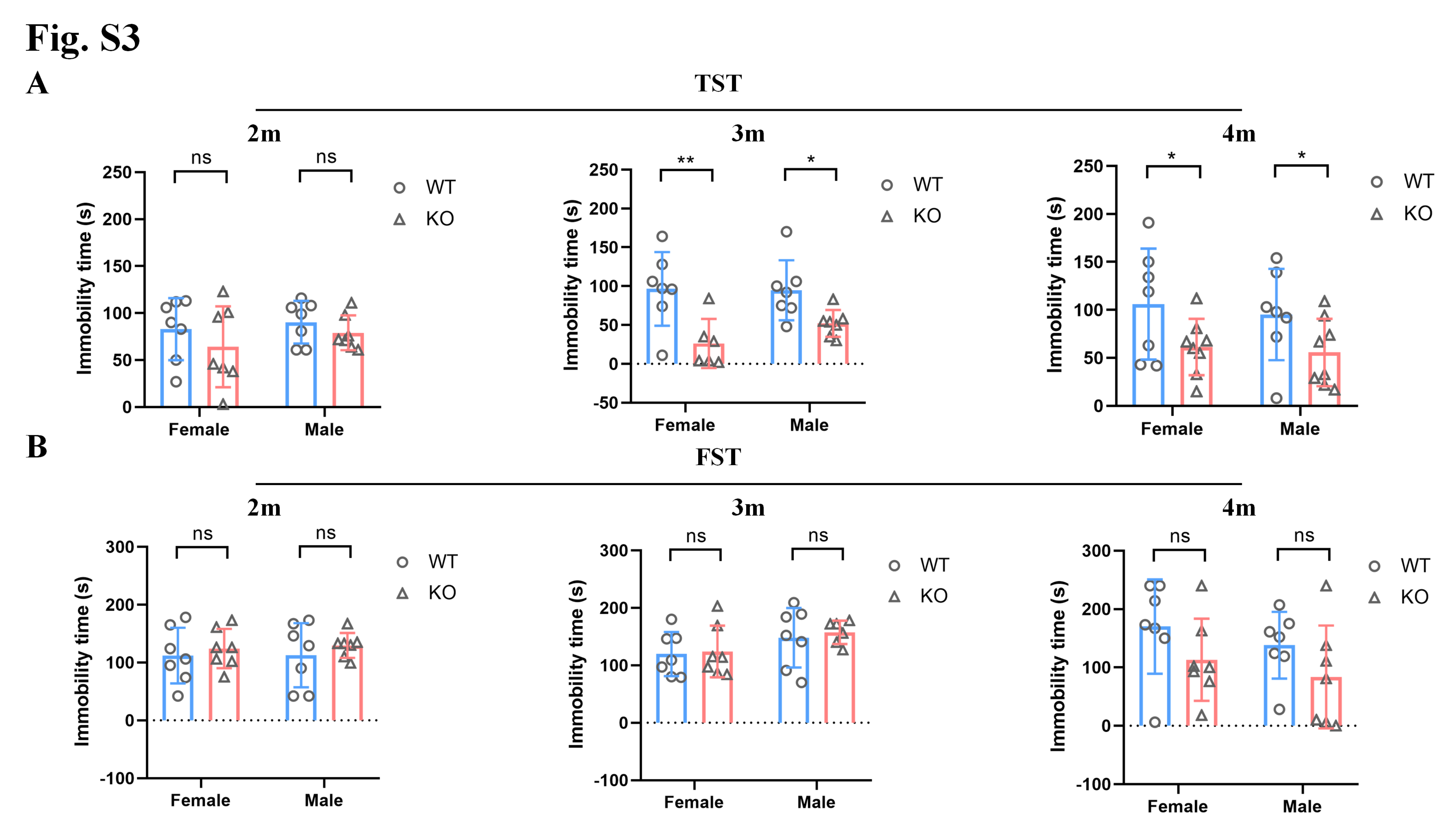
**

**Fig. S3 Depression-like behaviors in female and male Asm KO and WT mice. A.** The immobility time of mice at different ages in the TST. **B.** The immobility time of mice at different ages in the FST. All data are shown as mean±SD. * *p* < 0.05; ** *p* < 0.01; ns means no significance vs WT mice. *n*=6-8.

**
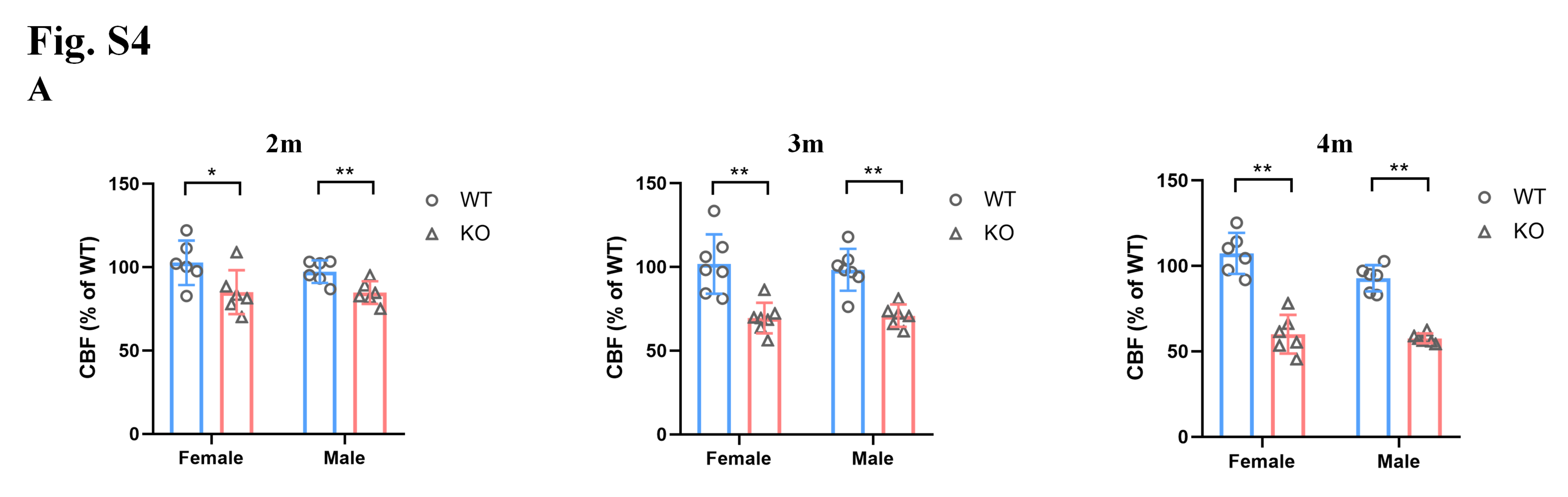
**

**Fig. S4 Cerebral blood flow in female and male Asm KO and WT mice.** The quantitative analysis of CBF obtained from laser speckle blood flow imager in mice at different ages. All data are shown as mean±SD. * *p* < 0.05; ** *p* < 0.01; ns means no significance vs WT mice. *n*=6-7.
